# Intravenous cyclophosphamide therapy for patients with severe ocular inflammatory diseases who failed other immunomodulatory therapies

**DOI:** 10.1186/s12348-023-00372-z

**Published:** 2024-03-11

**Authors:** Irmak Karaca, Elaine M. Tran, SungWho Park, Albert Bromeo, Hassan Khojasteh, Anh Ngọc Tram Tran, Negin Yavari, Amir Akhavanrezayat, Cigdem Yasar, Gunay Uludag Kirimli, Ngoc Tuong Trong Than, Muhammad Hassan, Christopher Or, Hashem Ghoraba, Diana V. Do, Quan Dong Nguyen

**Affiliations:** Spencer Center for Vision Research, Byers Eye Institute, Stanford University, Palo Alto, CA USA

**Keywords:** Cyclophosphamide, Scleritis, Uveitis, Severe, Refractory, Immunomodulatory therapy

## Abstract

**Background:**

Ocular inflammatory diseases, including scleritis and uveitis, have been widely treated with immunomodulatory therapies (IMTs) as a steroid-sparing approach. Such strategy includes conventional therapies (antimetabolites, alkylating agents, and calcineurin inhibitors) as well as biologic agents like adalimumab, infliximab, rituximab, and tocilizumab. Cyclophosphamide (CP) is an alkylating agent and mainly inhibits the functioning of both T and B cells. Though known to have potential adverse events, including bone marrow suppression, hemorrhagic cystitis, and sterility, CP has been shown to be efficacious, especially in recalcitrant cases and when used intravenous (IV) for a limited period.

**Main findings:**

We conducted a retrospective case-series to assess the safety and efficacy of CP therapy for patients with severe ocular inflammatory diseases who failed other IMTs. Medical records of 1295 patients who presented to the Uveitis Clinic at the Byers Eye Institute at Stanford between 2017 and 2022 were reviewed. Seven patients (10 eyes) who received CP therapy for ocular inflammatory diseases with at least one year of follow-up were included. The mean age of the patients (4 males, 3 females) was 61.6 ± 14.9 (43.0–89.0) years. Clinical diagnoses included necrotizing scleritis (5 eyes), peripheral ulcerative keratitis (2 eyes), orbital pseudotumor (1 eye), HLA-B27 associated panuveitis and retinal vasculitis (2 eyes). Ocular disease was idiopathic in 3 patients, and was associated with rheumatoid arthritis, IgG-4 sclerosing disease, dermatomyositis, and ankylosing spondylitis in 1 patient each. All the patients had history of previous IMT use including methotrexate (5), mycophenolate mofetil (3), azathioprine (1), tacrolimus (1), adalimumab (2), infliximab (4), and rituximab (1). The mean follow-up time was 34.4 ± 11.0 (13–45) months, and mean duration of CP therapy was 11.9 ± 8.8 (5–28) months. Remission was achieved in 5 patients (71.4%). Four patients (57.1%) experienced transient leukopenia (white blood cell count < 4000/mL).

**Short conclusion:**

CP therapy can be considered a potentially effective and relatively safe therapeutic option for patients with severe ocular inflammatory diseases who failed other IMTs including biologics (TNFa and CD20 inhibitors).

## Introduction

Cyclophosphamide (CP) is an alkylating agent and displays a cytotoxic effect on rapidly proliferating cells [1]. Its mechanism of action mainly inhibits the functioning of both T and B cells, resulting in suppression of the immune system [2]. CP was first introduced for the treatment of uveitis of unknown etiology in 1952 [3], and has since been utilized in the management of various types of ocular inflammatory diseases, including Adamantiades-Behçet’s disease [4], Vogt-Koyanagi-Harada (VKH) syndrome [5], ocular cicatricial pemphigoid (OCP) [6], and peripheral ulcerative keratitis (PUK) [7], as well as systemic autoimmune diseases like granulomatosis polyangiitis [8], rheumatoid arthritis [9], polyarteritis nodosa [10], and systemic lupus erythematosus [11]. Though known to have potential adverse events, including bone marrow suppression, hemorrhagic cystitis, and sterility, CP has been shown to be efficacious, especially in recalcitrant cases and when used intravenous (IV) for a limited period [12, 13].

In recent years, ocular inflammatory diseases, including scleritis and uveitis, have been widely treated with immunomodulatory therapies (IMTs) as a steroid-sparing approach. Such strategy includes conventional therapies (antimetabolites, alkylating agents, and calcineurin inhibitors) as well as biologic agents like adalimumab, infliximab, rituximab, and tocilizumab [14]. The Multicenter Uveitis Steroid Treatment (MUST) Trial and Follow-up Study has demonstrated that corticosteroid supplemented IMTs improved visual outcomes, controlled inflammation, and reduced macular edema compared with an intravitreal fluocinolone acetonide implant in patients with intermediate uveitis, posterior uveitis, or panuveitis [15]. In addition to successful use of conventional therapies, biologic agents have been increasingly used with promising results, especially in cases of ocular inflammation refractory to standard therapies [16]. Scleritis in particular is a potentially vision-threatening condition, and may also require IMTs, particularly when it presents as necrotizing scleritis or associated with systemic vasculitis [17]. Sainz de la Maza et al. [18] indicated that patients most often will respond to IMTs, mainly alkylating agents like CP, if the underlying vasculitis is potentially lethal. Given the varying severity of these entities, as well as challenges in treatment, management of these patients should be tailored to each patient, based on their clinical findings, associated underlying diseases, and safety profile of the agent.

Herein, we aimed to describe our experience on the efficacy and safety of IV CP in treating severe ocular inflammatory diseases in patients who failed with other IMTs including biologic agents.

### Materials and methods

Medical records of 1295 patients who presented to the Uveitis Clinic at the Byers Eye Institute at Stanford between 2017 and 2022 were retrospectively reviewed using the Stanford Research Repository (STARR) tool. Seven patients (10 eyes) who received CP therapy for severe ocular inflammatory diseases with at least one year of follow-up were identified and included in the study. Data review included demographics of patients, ocular findings, underlying systemic diseases, previous and concomitant therapies, total treatment duration, treatment outcomes, adverse events, and total follow-up time. Remission was defined as the absence of active ocular inflammation with or without therapy and was documented based on the clinical exam and imaging findings depending on the presence of scleral or intraocular inflammation. Any recurrence of ocular inflammation (i.e., anterior chamber or vitreous cells; inflammatory changes with redness on slit lamp examination; presence of vascular leakage of optic disc/vessels on fluorescein angiography), as well as associated comorbidities and complications during the follow-up period were also recorded.

The present study was approved by the Stanford University Institutional Review Board and followed the Helsinki Declaration tenets, the United States Code of Federal Regulations Title 21, and the Harmonized Tripartite Guidelines for Good Clinical Practice (1996). Each patient signed a written informed consent. SPSS 25.0 (SPSS Inc., Chicago, IL, USA) was used in the statistical analysis.

## Results

The mean age of the patients (4 male and 3 female) was 61.6 ± 14.9 (range, 43.0–89.0) years. Clinical diagnoses included necrotizing scleritis (5 eyes of 4 patients), peripheral ulcerative keratitis (2 eyes of 1 patients), orbital pseudotumor (1 eye of 1 patient), HLA-B27 associated panuveitis and retinal vasculitis (2 eyes of 1 patient). Ocular disease was idiopathic in 3 patients, and was associated with rheumatoid arthritis, IgG-4 sclerosing disease, dermatomyositis, and ankylosing spondylitis in 1 patient each (Table 1). All patients had history of previous IMT use. Previous IMTs included methotrexate (5 patients), mycophenolate mofetil (3 patients), azathioprine (1 patient), tacrolimus (1 patient), adalimumab (2 patients), infliximab (4 patients), and rituximab (1 patient). The mean total follow-up time was 32.6 ± 11.6 (13–45) months.

**Table 1:** Summary of characteristics and therapies of patients with ocular inflammatory diseases treated with intravenous cyclophosphamide

Case	Age (years), Sex	Total follow-up time (months)	Ocular disease	Underlying systemic disease	CP therapy duration	Side effects	Completion of therapy/flare-ups during or after therapy	Final therapy after CP	Previous IMTs
1	89, Female	32	Necrotizing scleritis, Right eye	Rheumatoid arthritis	11	Leukopenia, abdominal pain	Yes/No	IFX (due to ongoing systemic disease activity)	MTX, ADA, IFX
2	76, Female	40	Necrotizing scleritis, Right eye	IgG-4 sclerosing disease	5	None	Yes/No	MMF	MTX, RTX
3	65, Male	42	Necrotizing scleritis, Both eyes	None	28	Leukopenia, fatigue, elevated liver enzymes	No/Yes (recurrence of scleral inflammation 6 months after CP stopped; restarted)	N/A	MMF, IFX
4	70, Male	45	Orbital pseudotumor, Left eye	Dermatomyositis	6	Hematuria	Yes/No	MMF	MMF, IFX, Tacrolimus
5	43, Male	23	HLA-B27 associated panuveitis and retinal vasculitis, Both eyes	Ankylosing spondylitis	8	Transient fever	No/No	N/A	AZA, MMF, MTX, ADA, IFX
6	65, Female	13	Necrotizing scleritis, Left eye	None	13	Leukopenia	Yes/No	MTX	MTX
7	50, Male	42	Peripheral ulcerative keratitis, Both eyes	None	6	Leukopenia	Yes/No	MMF	MTX

*ADA* Adalimumab, *AZA* Azathioprine, *CP* Cyclophosphamide, *IFX* Infliximab, *MMF* Mycophenolate mofetil, *MTX* Methotrexate, *RTX* Rituximab

CP therapy was given as IV infusions at the dose of 1 g/m^2^ of body surface area every 3 to 4 weeks (pending schedules of the patients). IV CP infusions were preferred over oral CP given their rapid control of inflammation with better side effect profile [19]. At the time of CP initiation, all biologics were discontinued 4–6 weeks in advance. CP therapy was planned to be continued until the ocular inflammation got into remission. In addition, IV methylprednisolone (MP) at the dose of 750–1000 mg for 1–3 days monthly was given to augment the immunomodulatory effect of IV CP therapy on the day of IV CP infusion (1^st^ day) and the subsequent days (2^nd^ and 3^rd^ day of the month) accordingly. Patients were closely monitored for any possible adverse events. Baseline complete blood count and complete metabolic panel and urinalysis were performed and repeated every two months during the follow-up of each patient. White blood cell count (WBC) was monitored to guide dose adjustment, and was tolerated down to 3.0 to 4.5 × 10^9^ cells/L. In cases of lower WBC levels, CP therapy was held until the WBC count returned to normal range level. The patients were also hydrated adequately to wash out potential toxic metabolites from the urogenital system. Trimethoprim sulfamethoxazole prophylaxis was initiated (3 times weekly) to reduce the risk of opportunistic infections*.*

The mean duration of CP therapy was 10.9 ± 7.5 (range, 5–28) months. Median visual acuity (VA) at the initiation of IV CP therapy was 20/50 (range; 20/400 – 20/20). Inflammation improved in all patients. Complete remission was achieved in five patients (71.4%). One patient achieved resolution of disease but continues CP while waiting for surgical intervention, and another patient had a flare of his scleritis and was restarted on CP therapy, leading to stabilization of the disease. Four patients (62.5%) experienced leukopenia with the lowest WBC level at 2.2 × 10^9^ cells/L. Among them, the dose of CP therapy was required to be reduced to 750 mg/m^2^ of body surface area per month in one patient along with the use of pegfilgrastim during the CP infusions. One patient had to skip four CP infusions until his WBC recovered to normal range. Additionally, one patient had single episode of fever and one patient reported fatigue after the infusions; one patient experienced hematuria with subsequent unremarkable work-up, and one patient had brief self-limited episode of abdominal pain. One patient tolerated CP infusions without any adverse events. Among the six patients who successfully discontinued IV CP therapy during the follow-up, two patients had persistent borderline WBC levels with lack of opportunistic infections, whereas the adverse events were reversible in the remaining patients.

Following the discontinuation of IV CP therapy, four patients were successfully maintained on mycophenolate mofetil (2000 – 3000 mg/daily) and one patient on methotrexate (15 mg weekly) therapy. One patient was initiated on infliximab (10 mg/kg monthly) therapy given continued systemic disease activity.

Associated comorbidities and complications noted during the follow-up period included glaucoma in seven eyes of six (75%) patients which required tube shunt implantation in 3 (37.5%) eyes, scleral patch grafting in 3 (27.3%) eyes of two patients due to advanced scleral thinning, tarsorrhaphy in 2 (18.2%) eyes of two patients, and conjunctivoplasty in one (9.1%) eye of one patient. Visual acuity (VA) improved in 4 (36.4%) eyes of three patients and maintained in 5 (45.5%) eyes of four patients during the follow-up. Two eyes of two patients showed deterioration in VA due to vascularized cornea in one eye and cataract development in the other eye. Median VA at the final follow-up was 20/50 (range; 20/400 – 20/20).

## Discussion

The index study shows that IV CP therapy is an efficacious treatment choice with an acceptable safety profile in patients with severe ocular inflammatory diseases who failed with other IMTs including biologic agents. All patients in the study population responded to IV CP therapy, 71.4% of whom had complete remission of disease activity by the end of follow-up. This was especially conspicuous considering the presence of rapidly progressive, severe ocular inflammation and the failure with other IMTs including biologic agents. All patients had history of previous IMT use, and five (71.4%) of whom had received [3]1 biologic agent before initiation of IV CP. Two (25%) patients (3 eyes) had previously used both adalimumab and infliximab. One patient had scleritis which was refractory to rituximab but was finally controlled following 5 cycles of IV CP therapy. The most seen adverse event was decreased WBC levels, which was largely reversible in the study population. And though, in this setting of severe, destructive inflammation, the primary goal was preservation of the structural integrity of the globe, many patients benefited from stable, and even improved visual acuity.

Intravenous CP was first used in ophthalmic inflammation by Wong et al. [20] and then also shown to be effective in Adamantiades-Behçet’s disease [21] and severe refractory uveitis [22]. It has several advantages over oral CP, including faster control of severe ocular inflammation, usually transient (rather than permanent) leukopenia, and reduced risk of toxicity (as it bypasses the bladder), enabling larger doses [13]. Nonetheless, potential adverse events should be carefully considered when choosing patients to receive this therapy. In the index study, no patients clinically experienced sterility, developed cancer, nor had any fatalities related to IV CP use, though it should be noted that the follow up period was relatively short. However, as with making any clinical decision, risks must be weighed against the benefits, and in cases of severe, refractory vision-threatening inflammation, utilization of IV CP therapy should be considered, given the potential for achieving an otherwise elusive remission. And, as proposed by the European League against Rheumatism in order to decrease the risk of toxicity further [23] providers also have the option to switch to a maintenance regimen with a milder side effect profile once remission is achieved.

Biologic agents have been indicated as an effective treatment option for non-infectious ocular inflammation, including refractory uveitis and scleritis [24–26]. In a multicenter study of 34 patients with severe and refractory PUK, 48% of the patients treated with TNF-alpha inhibitors were required switching to another biologic agent, while no switching was needed in those receiving non-TNF-alpha agents like rituximab and tocilizumab [27]. Rituximab, a fully humanized monoclonal anti-CD20 antibody, has been demonstrated as an efficacious and safe therapy for refractory uveitis or scleritis with potential relapse of disease following discontinuation of treatment [28]. Sharma et al. [29] recently reported on an 8-year real-world prospective analysis showing that TNF-alpha inhibitors successfully induced sustained remission in 91% of patients with sustained steroid-sparing effect in 75% of the patients with uveitis or scleritis. On the other hand, 51% of patients experienced at least one relapse of inflammation during the follow-up. They emphasized that, despite success with TNF-alpha inhibitors, wider treatment options are necessary, as well as further assessment of which uveitic entities respond best, in order to optimize outcomes. Therefore, IV CP therapy should always be kept in our armamentarium as an alternative therapeutic option and may be utilized for a short term in order to achieve swift control of ocular inflammation, particularly in patients with severe, rapidly progressive disease which has failed with other IMTs.

The present study is informative as it demonstrates the efficacy of IV CP profile in the management of severe ocular inflammation in the modern era. There are some limitations of the study including the retrospective design, small sample size with relatively short follow-up period considering the adverse events like cancer development and associated mortality. In addition, as being the tertiary center setting, the patients usually had severe and resistant ocular inflammation. In addition, all patients received IV MP along with IV CP therapy during follow-up periods. Certainly, MP can also contribute to the improvement of the ocular inflammation. However, prior to IV CP therapy, patients were also receiving IV MP (at the similar dose of 750–1000 mg for 1–3 days monthly) during their therapy course with other biologics such as IFX and RTX. Thus, the confounding effect of MP would be irrelevant considering the patients had already failed with prior therapy. In addition, patients who were successfully discontinued IV CP therapy, were also discontinued IV MP therapy at the same time and continued to be followed up with antimetabolites in 5 patients (6 eyes) and IFX only in 1 patient (1 eye) (given active systemic disease). Considering short term pulse effect of IV MP therapy with no role in immunomodulation, this remission of disease may likely be attributed to IV CP therapy itself. Moreover, we have observed clinically that MP alone often does not lead to remission in eyes with severe or refractory ocular inflammation. Hence, we have employed IV CP and added IV MP to achieve synergistic effects as also previously described in the literature [30]. Importantly, long term treatment of these patients should not include high-dose corticosteroids, given its side effects.

In conclusion, IV CP therapy may be considered a potentially effective treatment option with a relatively acceptable safety profile for patients with severe ocular inflammatory diseases who failed with other IMTs including biologic agents (TNFa and CD20 inhibitors). Close monitoring with regular blood work is essential for the management of these patients, as leukopenia was the most common adverse event. Judicious use of IV CP appears favorable for severe, resistant ocular inflammation particularly when the potentially vision-saving benefits outweigh the substantial potential side effects of therapy.

## Data Availability

The datasets generated and analysed is available upon reasonable request and in compliance with local data protection policy.
